# A High-Potency Bovine Consensus Interferon Delivered by an Oral *Lactococcus lactis* System Exhibits Antiviral Activity Against VSV and BEV

**DOI:** 10.3390/ani16152327

**Published:** 2026-07-30

**Authors:** Hongzhe Zhao, Rongyan Li, Yang Yang, Yingying Ma, Yanping Jiang, Jiaxuan Li, Wen Cui, Xinyuan Qiao

**Affiliations:** Heilongjiang Key Laboratory for Animal Disease Control and Pharmaceutical Development, Department of Preventive Veterinary Medicine, College of Veterinary Medicine, Northeast Agricultural University, 600 Changjiang Road, Harbin 150030, China; hongzhezhao@163.com (H.Z.); lry15184755830@163.com (R.L.); yangyangneau@163.com (Y.Y.); mayingying@neau.edu.cn (Y.M.); jiangyanping8198@163.com (Y.J.); lijiaxuan.1993@163.com (J.L.); cuiwen_200@163.com (W.C.)

**Keywords:** BoIFN-α, consensus interferon, *L. lactis*, antiviral activity

## Abstract

Interferon-alpha (IFN-α) mediates innate antiviral immunity. Although it comprises multiple subtypes, their functional divergence and application potential remain incompletely characterized. In this study, we identified 13 BoIFN-α subtypes (A1–A13) in the bovine genome through bioinformatic analysis. Prokaryotic expression and functional comparison revealed substantial differences in antiviral activity among these subtypes (up to 77.7-fold), with BoIFN-A8 being the most potent. Based on these findings, we designed a novel consensus interferon, BoIFN-Acons, by multiple sequence alignment and successfully synthesized it. When expressed in *E. coli*, BoIFN-Acons exhibited superior antiviral potency (2.36 × 10^5^ U/mg), which was 16.2-fold higher than that of the most active natural subtype. To establish a safe delivery system, we integrated the codon-optimized gene into a food-grade *L. lactis* expression platform and achieved efficient supernatant production. In vitro assays confirmed that the recombinant BoIFN-Acons effectively suppressed replication of Vesicular stomatitis virus (VSV) and Bovine enterovirus (BEV) and significantly upregulated interferon-stimulated genes (ISGs) such as *Mx1* and *OAS1*. Furthermore, in vivo experiments demonstrated that oral administration of the engineered *L. lactis* strain alleviated virus-induced clinical symptoms, reduced viral loads in major tissues, and induced upregulation of immune-related genes in the spleen, showing significant prophylactic and therapeutic efficacy. Collectively, this study systematically elucidated the functional diversity of BoIFN-α subtypes and developed a highly active consensus interferon delivered via an oral lactic acid bacteria system, providing a new strategy for the eco-friendly control of animal viral diseases.

## Simple Summary

This study successfully developed a novel, high-potency bovine consensus interferon (BoIFN-Acons) delivered orally via a food-grade *L. lactis* system. Bioinformatic and functional analysis first revealed up to a 77.7-fold difference in antiviral activity among natural BoIFN-α subtypes. The engineered BoIFN-Acons dramatically outperformed them all, showing a 16.2-fold higher potency than the most active natural variant. When secreted by recombinant *L. lactis*, BoIFN-Acons exhibited strong antiviral activity against both VSV and BEV, two representative RNA viruses, significantly upregulating interferon-stimulated genes. Crucially, oral administration in a mouse model provided effective prophylactic and therapeutic protection, alleviating symptoms and reducing viral loads in major tissues. This work establishes a safe and efficient oral interferon delivery platform, offering a promising strategy for the eco-friendly control of bovine viral diseases.

## 1. Introduction

Type I interferons (IFNs) were first discovered in 1957 as soluble factors mediating viral interference [1]. Among them, IFN-α binds to the ubiquitous IFNAR receptor, activating the JAK-STAT pathway to upregulate hundreds of interferon-stimulated genes, thereby establishing a broad-spectrum antiviral state [2,3]. In humans, IFN-α is encoded by a multigene family whose members exhibit functional heterogeneity [4]. Similarly, in swine, the various IFN-α subtypes exhibit significant differences in antiviral activity, with only a few subtypes being highly potent [5]. These findings establish that functional diversification among IFN-α subtypes is a conserved mammalian feature, yet the most potent variants for therapeutic use often remain unidentified without systematic comparisons.

The *Bos taurus* genome harbors multiple IFN-α subtypes [6]. However, aside from limited characterizations of individual members such as BoIFN-α1, a systematic side-by-side comparison of their antiviral activities has never been reported [7]. Although a bovine consensus omega interferon (CoBoIFN-ω) has been successfully designed from 14 ω-subtypes with enhanced activity [8], the corresponding consensus strategy for the α-family remains unexplored.

Regarding production platforms, *E. coli* expression suffers from endotoxin contamination and inclusion body formation, while yeast and mammalian systems face challenges of aberrant glycosylation and high costs, respectively [9,10]. In contrast, the food-grade *L. lactis* NICE system, utilizing the safe inducer nisin, enables efficient secretion of bioactive proteins without antibiotic markers, offering a safe and orally deliverable alternative [11,12]. Recent studies have validated the feasibility of recombinant *L. lactis* for delivering antiviral proteins in veterinary settings [13].

Given the above, three critical gaps persist: (i) the antiviral potency hierarchy among bovine IFN-α subtypes remains unknown; (ii) a consensus interferon for the bovine α-family has not been developed; and (iii) an oral delivery platform for bovine IFN-α is lacking. To address these, we systematically compared all BoIFN-α subtypes, designed a novel BoIFN-Acons preserving key structural motifs, and achieved its secretory expression in *L. lactis*. We then evaluated its antiviral efficacy against VSV and BEV in vitro and in a murine model. Our work reveals the functional diversity of bovine IFN-α subtypes, validates the consensus design strategy for generating a hyperactive variant, and demonstrates the potential of an oral *L. lactis* delivery system, providing a viable path toward practical antiviral interventions in livestock.

## 2. Materials and Methods

### 2.1. Acquisition and Characterization of BoIFN-α Family Gene Sequences

Thirteen BoIFN-α subtype genes (designated A1–A13) were identified from the *Bos taurus* UMD 3.1.1 genome by BLAST (version 2.15.0) search using known BoIFN-α sequences as templates. After multiple sequence alignment (MegAlign, DNASTAR Inc., Madison, WI, USA) and signal peptide prediction (SignalP 5.0, DTU Health Tech, Kongens Lyngby, Denmark), three pseudogenes (A2, A11, and A12) were excluded, leaving ten BoIFN-α subtypes for further study.

### 2.2. Amplification, Vector Construction, and Expression of BoIFN-α Genes

Total genomic DNA was isolated from 200 mg of fresh bovine liver tissue with a commercial kit (Solarbio, Beijing, China), followed by PCR amplification of BoIFN subtypes. This process involved an initial round using outer-specific primers (Table S1) and a subsequent nested round employing mature peptide-specific primers (Table S2) for each subtype. The PCR products were first cloned into the pMD-19T simple vector and subcloned into the pET-28a-SUMO expression vector. The BoIFN-Acons amino acid sequence was designed using EMBOSS Cons (https://www.ebi.ac.uk/jdispatcher/msa/emboss_cons, accessed on 20 April 2024) by scanning 13 BoIFN-α subtype sequences (including pseudogenes) and selecting the most frequently occurring amino acid at each position. Key structural elements were preserved, including the five α-helical frameworks (A–E) critical for receptor binding, the disulfide bonds Cys1-Cys99 essential for antiviral activity, and Cys29-Cys139 associated with thermal stability, to maintain protein structural and functional integrity. The corresponding gene, encoding a C-terminal FLAG-tag, was synthesized by Sevgen Biotech (Beijing, China) and cloned into the pET-28a-SUMO vector using the *Bam*HI and *Xho*I restriction sites. The recombinant plasmid was transformed into *E. coli* Rosetta and protein expression was induced with 1 mM IPTG at 16 °C for 8 h. Cells were harvested, resuspended in PBS, and lysed by sonication, and the total protein concentration in the clarified supernatant was measured.

### 2.3. Assessment of the Antiviral Activity of BoIFN-Acons

The antiviral activities of the BoIFN-α subtypes and the BoIFN-Acons were determined in MDBK cells using a cytopathic effect (CPE) reduction assay against VSV. Confluent monolayers of MDBK cells in 96-well plates were incubated with serial twofold dilutions of each IFN sample in maintenance medium (5% FBS DMEM) for 16 h at 37 °C and 5% CO_2_. Cell control wells (medium only) and virus control wells (no IFN) were included on the same plate. After incubation, the medium was aspirated, and the cells were washed with PBS. The cells were then inoculated with approximately 100 TCID_50_ of VSV diluted in 2% FBS DMEM, cell control wells received medium only. Plates were incubated until complete CPE observed in the virus control wells. Cells were fixed with 10% methanol and stained with 0.1% crystal violet solution. The dye bound to surviving cells was solubilized with destaining solution, and absorbance was measured at 570 nm. Protective efficacy of each IFN was calculated, and the titer was expressed as the reciprocal of the dilution that conferred 50% protection from viral CPE, as calculated by the Reed-Muench method. The specific activity values (U/mg) were calculated based on total soluble protein in clarified bacterial lysates and are presented for relative potency comparison among subtypes, rather than as absolute specific activities of purified proteins.

### 2.4. Expression of BoIFN-Acons in L. lactis

The gene encoding BoIFN-Acons was designed for secretory expression in the food-grade host *L. lactis* NZ3900 using the NICE system. The expression cassette included the Usp45 signal peptide, the LEISSTCDA propeptide, the mature BoIFN-Acons sequence, and a C-terminal FLAG tag. The cassette was synthesized and cloned into the pNZ8149 vector to generate the recombinant plasmid pNZ-BoIFN-Acons. This construct was first assembled in an *E. coli* cloning vector by homologous recombination, sequence-verified, and then subcloned into pNZ8149 using *Noc*I and *Sac*I restriction sites. The resulting recombinant plasmid was electroporated into competent *L. lactis* NZ3900 cells, and positive clones carrying pNZ-BoIFN-Acons were selected and confirmed by PCR and DNA sequencing. For protein expression, a positive clone was cultured in GM17 medium to an OD_600_ of 0.4, and expression was induced with 100 ng/mL nisin. To optimize production, various nisin concentrations (40–180 ng/mL) and induction times (2–8 h) were tested. After induction, the recombinant BoIFN-Acons protein was harvested from the culture supernatant by ammonium sulfate precipitation, followed by dialysis against PBS. To verify the secretory expression of the target protein, we performed Western blot analysis using an anti-FLAG monoclonal antibody.

### 2.5. Determination of the Antiviral Activity of the Recombinant L. lactis pNZ-BoIFN-Acons/NZ3900 In Vitro

The in vitro antiviral activity of the recombinant *L. lactis* strain pNZ-BoIFN-Acons/NZ3900 was comprehensively evaluated. Conditioned medium (CM) from the induced culture was collected, pH-adjusted, filter-sterilized, and quantified for total protein. A CCK-8 assay on MDBK cells was performed to determine the non-cytotoxic dilution for subsequent experiments. The direct antiviral activity of BoIFN-Acons-containing CM was evaluated against VSV and BEV using a standard CPE inhibition assay in MDBK cells, with CM from the empty vector (pNZ8149/NZ3900) serving as the control. To investigate temporal effects, pre- and post-treatment protocols were employed. For pre-treatment, MDBK cells were incubated with the CM for 16–18 h before infection with VSV (10 TCID_50_). For post-treatment, cells were first infected with VSV for 6–8 h and then treated with CM. In both setups, viral titers in the supernatant were determined by the TCID_50_ method at 12, 24, and 36 h post-infection. Finally, the ability of CM to induce ISGs was analyzed by qRT-PCR. MDBK cells were treated with serial dilutions of CM for 8 h. Total RNA was extracted and reverse-transcribed, and the relative mRNA expression of *Mx1*, *OAS1*, *ISG15*, and *IFITM1* was quantified using the 2^−ΔΔCt^ method with *GAPDH* as the reference gene.

### 2.6. Physicochemical Stability of Recombinant BoIFN-Acons

The physicochemical stability of BoIFN-Acons in conditioned medium from recombinant *L. lactis* was evaluated. To assess pH stability, the supernatant was adjusted to pH 2.0, 7.0, and 10.0 and incubated at 4 °C for 24 h, then neutralized. For thermal stability, aliquots were treated at 4 °C or 63 °C for 2 h. After these treatments, residual antiviral activity of each sample was determined by the CPE inhibition assay against VSV. Samples treated at pH 7.0 and 4 °C served as the respective controls.

### 2.7. Analysis of Antiviral Effects Following Oral Administration of Recombinant L. lactis pNZ-BoIFN-Acons/NZ3900

To evaluate the antiviral efficacy of orally administered recombinant *L. lactis*, specific pathogen free female BALB/c mice (6 weeks old, body weight 18–22 g) without any visible abnormalities were enrolled and randomly divided into six groups (*n* = 6 per group): blank control, VSV infected control, pNZ-BoIFN-Acons/NZ3900 preventive, pNZ-BoIFN-Acons/NZ3900 therapeutic, and their corresponding empty vector control groups. The preventive groups received oral bacterial suspensions (1 × 10^9^ CFU/mL) once daily for 5 days prior to VSV challenge (10^6^ TCID_50_ units per mouse in 100 μL PBS, a dose determined by preliminary experiments to induce consistent clinical signs without mortality), whereas the therapeutic groups were given the same suspensions for 5 days after the same viral challenge (Table 1). Body weight and clinical signs were recorded daily. At 3 and 6 days post infection, three mice from each group were euthanized, and viral loads in brain and lung tissues were determined by qPCR.

### 2.8. ISGs Induction by Recombinant L. lactis

To assess the induction of ISGs by oral administration of the recombinant strain, an additional 24 SPF female BALB/c mice (6 weeks old, weighing 18–22 g) were randomly divided into three groups (*n* = 8 per group) after a 1-week acclimatization. Mice in Group 1 received 100 μL (1 × 10^9^ CFU) of pNZ-BoIFN-Acons/NZ3900 bacterial suspension once daily by intragastric gavage; Group 2 received the same volume of the empty-vector pNZ8149/NZ3900 suspension; and Group 3 received PBS as a vehicle control. At days 0, 2, 4, and 6 post-treatment, two mice from each group were randomly selected and euthanized at each time point, and their spleens were aseptically harvested. Total RNA was extracted from spleen tissues, reverse-transcribed into cDNA, and subjected to quantitative real-time PCR using mouse *β-actin* as the internal reference gene. The relative mRNA expression levels of *OAS1* and *ISG15* were calculated using the 2^−ΔΔCt^ method.

### 2.9. Statistical Analysis

All data in this study were obtained from at least three independent experiments and are presented as mean ± SD. Comparisons among multiple groups were analyzed using one-way ANOVA followed by Tukey’s multiple comparison test, while comparisons between two groups were performed using two-tailed Student’s *t*-test, both using SPSS Statistics 22.0. Differences were considered significant at * *p* < 0.05, ** *p* < 0.01, *** *p* < 0.001, and **** *p* < 0.0001.

## 3. Results

### 3.1. Basic Information on the Bovine Interferon Family

A BLAST search against the *Bos taurus* UMD 3.1.1 reference genome database at NCBI identified 13 members of the BoIFN-α family (Table 2). Ten were classified as functional genes, while three (A2, A11, and A12) were identified as pseudogenes. The members were designated A1 through A13 according to their sequential order along the chromosome. SignalP 5.0 analysis revealed that all 13 BoIFN-α amino acid sequences contain a 23-amino acid signal peptide, followed by a 166-amino acid mature polypeptide (Figure S1).

### 3.2. The Anti-VSV Activity of Bovine Interferon Subtypes Varied Significantly in MDBK Cells, with BoIFN-Acons Being the Most Potent

**Figure 1:**
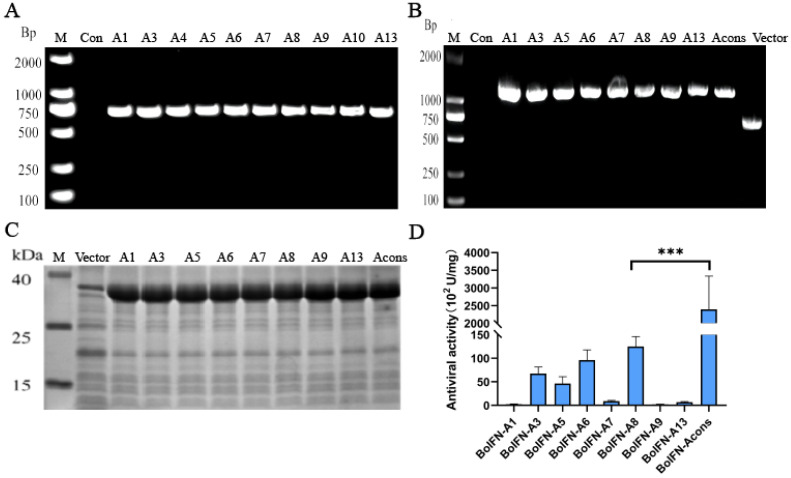
Amplification, prokaryotic expression, and activity validation of BoIFN-α family genes. (**A**) The recombinant clones containing BoIFN-α genes were identified by PCR. (**B**) PCR identification results for expression plasmids of BoIFN-α family genes. (**C**) The recombinant BoIFN-α subtypes were successfully expressed. (**D**) Antiviral activity of BoIFN-A1/3/5/6/7/8/9/13/cons. Data are presented as mean ± SD (*n* = 3 independent experiments). Differences were considered significant at *** *p* < 0.001.

The mature peptide-encoding genes of each BoIFN-α subtype were cloned into the pMD-19T vector. Colony PCR with universal primers confirmed the presence of specific bands of approximately 700 bp in all recombinant clones (Figure 1A), which were verified by sequencing. However, sequencing revealed frameshift mutations in the BoIFN-α A4 and A10 subtypes. Although these subtypes were annotated as intact genes in the reference genome, the frameshifts were identified in the amplicons from our bovine tissue sample. Given that frameshift mutations disrupt the downstream open reading frame and abrogate the structural domains essential for IFN-α bioactivity, these two variants were excluded from all subsequent expression and activity assays. Other bovine interferon subtypes were inserted into the pET-28a-SUMO vector. PCR screening with universal vector primers revealed that recombinant clones produced specific bands approximately 1500 bp (Figure 1B), which also matched the expected sizes and sequencing results. After induction of expression in the recombinant strains pET-28a-SUMO-BoIFN-A1, 3, 4, 5, 6, 7, 8, 9, 10, 13, and cons in Rosetta host cells, bacterial cultures were collected, ultrasonicated, and centrifuged. The resulting supernatants were analyzed by SDS-PAGE, which revealed that all recombinant strains expressed soluble 31 kDa BoIFN-α subtype proteins (Figure 1C). Finally, the antiviral activities of recombinant BoIFN-A1, 3, 5, 6, 7, 8, 9, 13, and cons were evaluated using a CPE inhibition assay. The results indicated distinct antiviral activities among the subtypes. BoIFN-A8 had the highest relative antiviral activity on MDBK cells (1.46 × 10^4^ U/mg), whereas BoIFN-A9 had the lowest (1.88 × 10^2^ U/mg). Notably, the relative antiviral activity of BoIFN-Acons (2.36 × 10^5^ U/mg) was significantly higher than all other subtypes (Figure 1D) (Table 3).

### 3.3. Efficient Secretion of BoIFN-Acons Was Achieved in L. lactis

**Figure 2:**
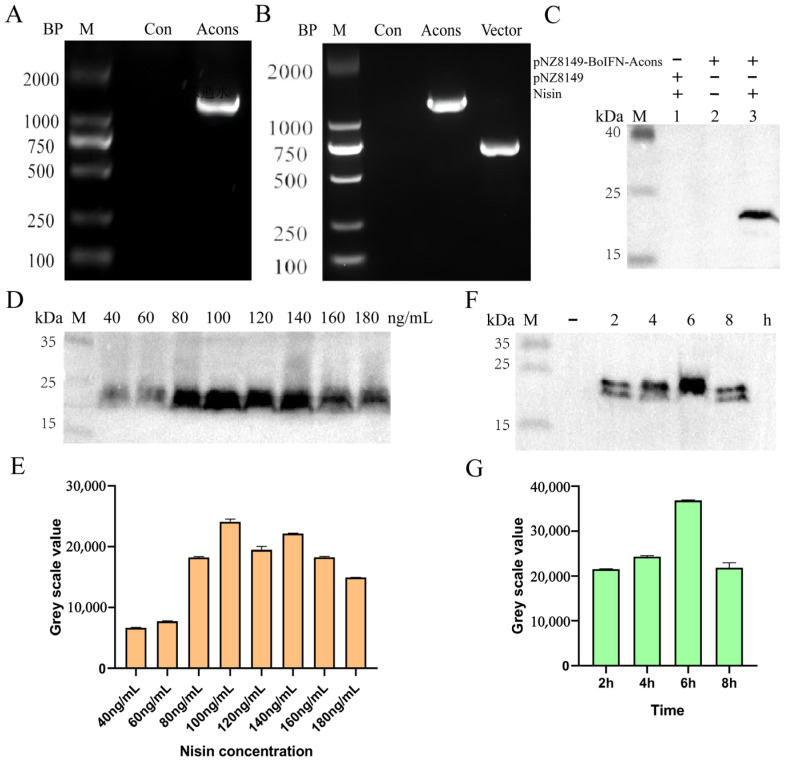
Identification of BoIFN-Acons expression in *L. lactis* NZ3900. (**A**) PCR verification results for the recombinant plasmid pET-28a-SUMO-Acons. (**B**) PCR verification results for the recombinant plasmid pNZ-BoIFN-Acons. (**C**) Western blot analysis confirmed the expression of BoIFN-Acons in NZ3900. (**D**) Effect of Different nisin Concentrations on the Secretory Expression of pNZ-BoIFN-Acons/NZ3900. (**E**) Analysis of the grey scale values in (**D**). (**F**) Optimisation of pNZ-BoIFN-Acons/NZ3900 induction time. (**G**) Analysis of the grey scale values in (**F**). Data are presented as mean ± SD (*n* = 3 independent experiments).

The recombinant plasmid pET-28a-SUMO-Acons was initially verified by colony PCR using pET-28a-SUMO-specific primers, which yielded a specific band of approximately 1500 bp, consistent with the expected size of the target gene (Figure 2A). The BoIFN-Acons gene was then ligated into the pNZ8149 vector to construct the recombinant plasmid pNZ8149-BoIFN-Acons. PCR confirmation using pNZ8149-specific primers produced a 1500 bp fragment (Figure 2B), and subsequent sequencing confirmed the correct insertion. For heterologous expression, *L. lactis* NZ3900 harboring either pNZ-BoIFN-Acons or the empty pNZ8149 vector were induced. Western blot analysis of the culture supernatant revealed a specific band at approximately 20 kDa in the induced pNZ-BoIFN-Acons/NZ3900 strain, matching the predicted molecular weight of BoIFN-Acons. No corresponding band was detected in the empty vector control or the uninduced culture, demonstrating successful secretory expression of BoIFN-Acons by the recombinant strain (Figure 2C). To optimize induction conditions, the recombinant strain pNZ-BoIFN-Acons/NZ3900 was induced with nisin at concentrations ranging from 40 to 180 ng/mL for 5 h. Western blot analysis of the supernatants indicated that the highest expression level was achieved at 100 ng/mL nisin (Figure 2D,E). A time-course induction using 100 ng/mL nisin was then performed. Samples collected at 2, 4, 6, and 8 h post-induction showed that protein yield peaked after 6 h of induction (Figure 2F,G). In addition, the genetic stability of the recombinant strain was confirmed by repeated freeze–thaw recovery from glycerol stocks, with PCR and Western blot analyses consistently verifying plasmid integrity and protein expression, indicating satisfactory stability under routine handling. However, systematic long-term evaluation will be required for industrial application.

### 3.4. Recombinant BoIFN-Acons from L. lactis Significantly Inhibited Viral Replication In Vitro, Exhibiting Efficacy in Both Preventive and Therapeutic Models

**Figure 3:**
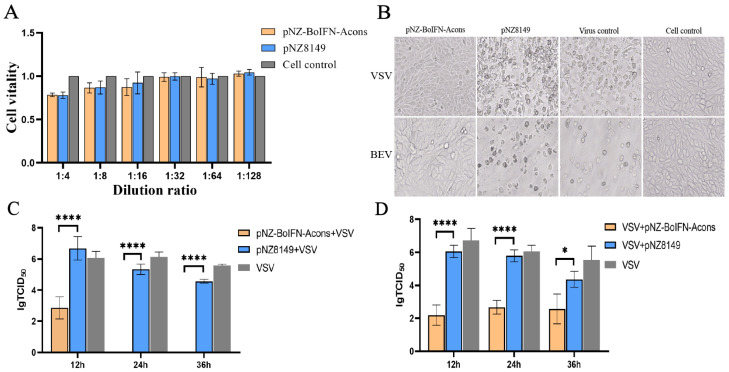
Identification of BoIFN-Acons as an inhibitor of VSV and BEV replication. (**A**) The effects of pNZ-BoIFN-Acons and pNZ8149 on the viability of MDBK cells. (**B**) Both BoIFN and Acons can inhibit lesions induced by VSV and BEV. (**C**) BoIFN-Acons can prevent VSV-induced infection. (**D**) BoIFN-Acons can treat infections caused by VSV. Data are presented as mean ± SD (*n* = 3 independent experiments). Differences were considered significant at * *p* < 0.05 and **** *p* < 0.0001.

The potential cytotoxicity of the culture supernatants from pNZ-BoIFN-Acons/NZ3900 and the control strain (pNZ8149/NZ3900) on MDBK cells was evaluated using the CCK-8 assay. Results indicated no significant difference in cell viability between the two groups across the tested concentrations. At dilution factors greater than 1:32, neither supernatant exhibited detectable cytotoxic effects; thus, a 1:32 dilution was used for all subsequent experiments (Figure 3A). The antiviral activity of the pNZ-BoIFN-Acons/NZ3900 culture supernatant was quantified against VSV and BEV using a CPE inhibition assay. The calculated antiviral titers reached 7.66 × 10^4^ U/mL for VSV and 4.47 × 10^4^ U/mL for BEV, respectively. Notably, cells treated with the BoIFN-Acons-containing supernatant were completely protected from virus-induced CPE, demonstrating that the *L. lactis*-expressed BoIFN-Acons significantly inhibited the replication of both viruses in MDBK cells (Figure 3B). To evaluate preventive efficacy, MDBK cells were pretreated with supernatants from pNZ-BoIFN-Acons/NZ3900 or the control for 16–18 h before infection with VSV (10 TCID_50_/0.1 mL). Viral titers in the culture media, collected at 12, 24, and 36 h post-infection (hpi), were determined. The group pre-treated with pNZ-BoIFN-Acons/NZ3900 supernatant showed a significant reduction in viral titer compared with controls (Figure 3C), confirming that BoIFN-Acons conferred effective protection against subsequent VSV challenge. The therapeutic potential was investigated by applying the supernatants to MDBK cells at 8 h post-infection with VSV. Viral titers were measured at 12, 24, and 36 hpi. Treatment with pNZ-BoIFN-Acons/NZ3900 supernatant significantly suppressed the increase in viral titer within infected cells (Figure 3D), providing evidence of a robust therapeutic effect of BoIFN-Acons on VSV-infected cells.

### 3.5. BoIFN-Acons Significantly Upregulated the Expression of ISGs in MDBK Cells

**Figure 4:**
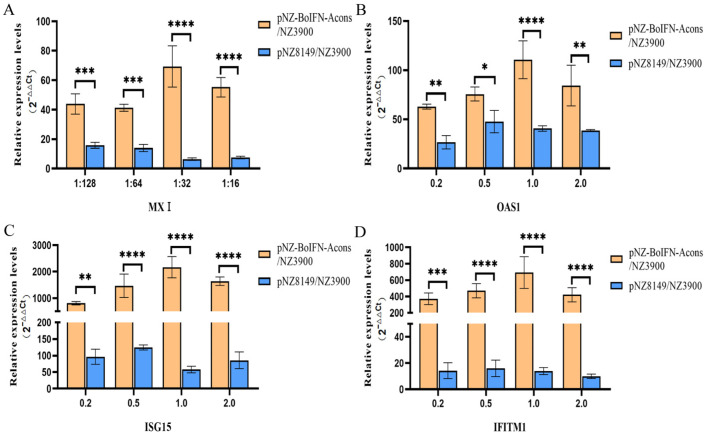
Effects of BoIFN-Acons treatment on the expression levels of immunological factor genes in cells. (**A**) *MX1*. (**B**) *OAS1*. (**C**) *ISG15*. (**D**) *IFITM1*. Data are presented as mean ± SD (*n* = 3 independent experiments). Differences were considered significant at * *p* < 0.05, ** *p* < 0.01, *** *p* < 0.001, and **** *p* < 0.0001.

The induction of ISGs by BoIFN-Acons was evaluated in MDBK cells treated with diluted culture supernatants. Quantitative RT-PCR analysis revealed that pNZ-BoIFN-Acons/NZ3900 supernatant significantly upregulated the expression of *Mx1*, *OAS1*, *ISG15*, and *IFITM1*. The peak induction was observed at a 1:32 dilution (Figure 4). The increase in ISG expression was highly significant (*p* < 0.01) compared to the control supernatant treatment.

### 3.6. Oral Delivery of Recombinant L. lactis Expressing BoIFN-Acons Confers Antiviral Protection by Inducing ISG Expression

**Figure 5:**
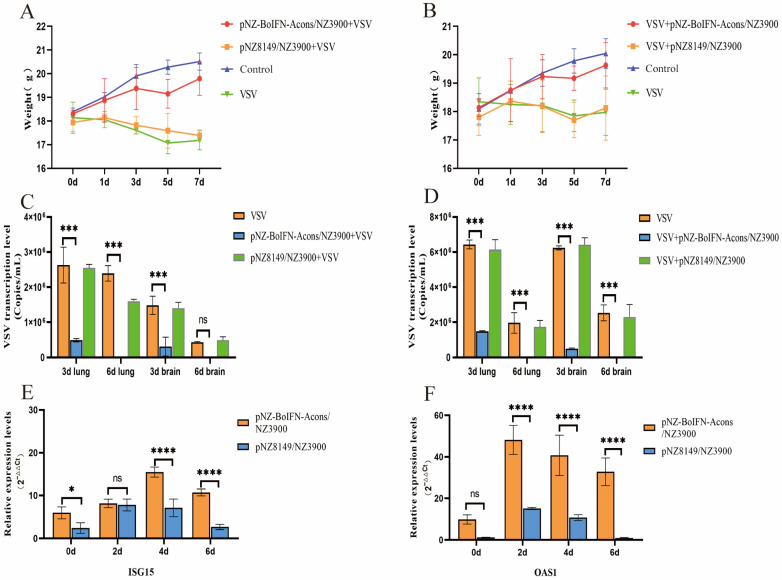
Evaluation of the In Vivo Antiviral Effects of Oral Recombinant Lactobacillus pNZ-BoIFN-Acons/NZ3900. (**A**) Body weight changes in mice in the prevention group. (**B**) Changes in body weight of mice in the treatment group. (**C**) Results of VSV gene copy number in the lungs and brains of mice in the prevention group. (**D**) Results of VSV gene copy number in the lungs and brains of mice in the treatment group. (**E**) Relative expression of the *ISG15* gene in mouse spleens. (**F**) Relative expression of the *OAS1* gene in mouse spleens. Data are presented as mean ± SD (*n* = 3 independent experiments). Differences were considered significant at * *p* < 0.05, *** *p* < 0.001, and **** *p* < 0.0001. ns—not significant.

Oral administration of recombinant *L. lactis* pNZ-BoIFN-Acons/NZ3900 significantly alleviated clinical symptoms and body weight loss induced by VSV challenge in mice (Figure 5A,B). The qPCR analysis revealed that pretreatment or postinfection treatment with pNZ-BoIFN-Acons/NZ3900 substantially reduced VSV mRNA levels in lung and brain tissues (Figure 5C,D). Concurrently, expression of the *ISG15* and *OAS1* was significantly upregulated in the spleen (Figure 5E,F). Collectively, these results demonstrate that the recombinant strain conferred effective prophylactic and therapeutic protection against VSV infection in vivo.

## 4. Discussion

Interferons are essential components of host defense, mediating immunomodulation, antiviral responses, and antitumor activity. Among type I interferons, IFN-α serves as a key initiator of innate antiviral immunity, and multiple subtypes exist in various species. In this study, we identified 13 BoIFN-α gene sequences (A1–A13) from the bovine reference genome, all encoding a 23-amino acid signal peptide and a 166-amino acid mature region. Although previous reports have noted functional diversity among IFN subtypes, with NK cell activation capacity also varying considerably [14,15], the specific activity profiles of bovine IFN-α family members had not been systematically compared. To address this gap, we expressed eight functional BoIFN-α subtypes in *E. coli* and evaluated their antiviral potencies. Our results revealed substantial variation, with BoIFN-A8 showing the highest activity (1.46 × 10^4^ U/mg) and BoIFN-A9 the lowest (1.88 × 10^2^ U/mg)—a 77.7-fold difference. The substantial functional divergence among BoIFN-α subtypes is consistent with recent comparative genomic studies. Peters et al. [6] demonstrated that Bovidae species have undergone lineage-specific expansions and contractions of IFN-α gene families, resulting in a larger and more diverse interferon repertoire than that of humans or mice. This expansion likely reflects evolutionary adaptation to the diverse viral pathogens encountered by ruminants [6]. The 77.7-fold difference in antiviral activity between BoIFN-A8 and BoIFN-A9 highlights the functional impact of this genetic diversity, suggesting that differential subtype potencies correspond to distinct roles in host antiviral defense. Similarly, swine IFN-α subtypes exhibit up to 100-fold activity differences, with IFN-α2, -α5, -α9, and -α10 being the most potent [5]. Collectively, these observations indicate that IFN-α subtype diversification coupled with functional specialization may represent a common evolutionary strategy across mammals for fine-tuning antiviral responses against a broad spectrum of pathogens.

To harness the favorable properties of multiple subtypes, we designed a consensus sequence, BoIFN-Acons, by multiple sequence alignment of all 13 subtypes, while preserving critical structural elements (α-helices A–E and the disulfide bonds Cys1-Cys99 and Cys29-Cys139) that are essential for receptor binding and biological activity. When expressed in *E. coli*, BoIFN-Acons exhibited an antiviral activity 16.2-fold higher than that of the most potent natural subtype (BoIFN-A8), confirming the success of the consensus strategy. This approach has been similarly effective in other species, including human IFN-αCon1 and porcine CoPoIFN-α [16,17]. More relevantly, Gao et al. designed a bovine consensus omega interferon (CoBoIFN-ω) by aligning 14 BoIFN-ω subtypes and selecting the most frequent amino acid at each position [8]. When expressed in Pichia pastoris, CoBoIFN-ω exhibited 3.94-fold and 14.3-fold higher antiviral activity against VSV than BoIFN-ω24 and BoIFN-ω3, respectively, and demonstrated efficacy against multiple bovine viruses including BEV, BHV-1, and BPIV3 through the JAK-STAT pathway [8]. Notably, our BoIFN-Acons, designed from 13 BoIFN-α subtypes (including pseudogenes), achieved a 16.2-fold improvement over the most potent natural subtype—a magnitude of enhancement comparable to that reported for CoBoIFN-ω. The consistency of these findings across different interferon families validates the consensus design strategy as a robust approach for generating hyperactive interferon variants with broad-spectrum antiviral potential.

While *E. coli* enables high protein yields, it poses risks of endotoxin contamination and inclusion-body formation; yeast systems may cause aberrant glycosylation, and mammalian systems are costly and unstable [9,10]. Therefore, we selected the food-grade *L. lactis* pNZ8149/NZ3900 system, which lacks spores and toxins, uses nisin as a safe inducer, and eliminates the need for antibiotic resistance markers [11]. To improve secretion, we incorporated the LEISSTCDA propeptide [18]. Under optimized conditions (100 ng/mL nisin, 6 h induction), BoIFN-Acons was efficiently secreted into the culture supernatant, as confirmed by Western blot. In addition, the physicochemical stability assays were performed using a self-controlled design, as no commercial bovine IFN-α reference standard is currently available. Notably, BoIFN-Acons retained 47–57% of its original activity (3.6–4.4 × 10^4^ U/mL) even under extreme conditions (pH 2.0, pH 10.0, and 63 °C), confirming its satisfactory stability. Therefore, all subsequent in vitro and in vivo antiviral evaluations were performed using the BoIFN-Acons secreted by this recombinant *L. lactis* strain. Conditioned medium from the recombinant strain showed no cytotoxicity at a 1:32 dilution and exhibited potent antiviral activity against both VSV and BEV, with titers of 7.66 × 10^4^ U/mL and 4.47 × 10^4^ U/mL, respectively. In both pre- and post-infection treatment protocols, the recombinant supernatant significantly reduced viral titers in MDBK cells, demonstrating both prophylactic and therapeutic efficacy. Mechanistically, BoIFN-Acons strongly upregulated ISGs, including *Mx1*, *OAS1*, *ISG15*, and *IFITM1*, in a dose-dependent manner. Type III interferons (IFN-λ) have recently been recognized for their distinct roles in mucosal antiviral defense. However, their efficacy is cell-type-dependent: bovine IFN-α, but not IFN-λ3, cleared BVDV in BTu primary epithelial cells due to low expression of the IFN-λ receptor IL-28Rα [19]. By contrast, IFN-α utilizes the ubiquitously expressed IFNAR receptor, enabling broad antiviral coverage across diverse tissues. Our finding that BoIFN-Acons induces potent ISGs upregulation in vitro and splenic ISGs expression in vivo further supports the advantage of IFN-α-based therapeutics for both local and systemic applications. In a mouse model, oral administration of the recombinant *L. lactis* strain significantly alleviated body weight loss and clinical signs, reduced VSV RNA loads in lung and brain tissues, and elevated splenic expression of *ISG15* and *OAS1*, confirming its protective effects in vivo. These findings align with a previous study using the same system to deliver salmon IFN-γ, further supporting the feasibility of oral lactic-acid-bacteria-based immunotherapy [13]. Beyond its antiviral effects, the oral *L. lactis* platform offers practical advantages for veterinary use: it enables mass administration via feed or water, avoids injection-related risks and costs, and its GRAS status eliminates concerns about endotoxins and antibiotic resistance markers—addressing key regulatory considerations. The recent success of recombinant *L. lactis* expressing human LL-37 in piglets and chickens further demonstrates the feasibility of this approach [20]. Collectively, these attributes support the translational potential of our strain for field applications. Given that interferons have broad immunomodulatory functions—such as promoting γδ T cell responses in mastitis and sensitizing cancer cells to NK cell-mediated killing—the capacity of BoIFN-Acons to elicit additional immune effects, including NK and T cell activation, presents a compelling direction for future research alongside its direct antiviral properties [21,22].

Our work builds upon prior patents (CN103450355B for a bovine consensus IFN-α and 201210491070 for oral interferon delivery via lactic acid bacteria) but advances the technology by: (i) systematically comparing all 13 bovine IFN-α subtypes and designing a novel consensus sequence; (ii) optimizing codon usage and GC content (from 59.6% to 38.4%) for *L. lactis* expression; (iii) enhancing secretion via the LEISSTCDA propeptide; and (iv) demonstrating both prophylactic and therapeutic in vivo efficacy. Related intellectual property is currently under application.

We acknowledge several limitations. First, the antiviral activity data for *E. coli*-expressed subtypes were obtained from crude lysates, and the U/mg values represent relative potencies based on total soluble protein rather than absolute specific activities of purified proteins. Future studies using affinity-purified BoIFN-Acons will be required to determine its true specific activity. Second, the in vivo protection data were generated exclusively in a murine surrogate model, which does not fully recapitulate the natural mucosal infection routes or digestive physiology of cattle. Therefore, homologous virus challenge studies in the target species, along with dose titration and long-term safety evaluations, are necessary before clinical translation. Third, the current antiviral evaluation was primarily focused on a limited panel of viruses; to further strengthen the veterinary applicability, forthcoming work will extend the antiviral activity testing to other economically significant bovine pathogens, including lumpy skin disease virus (LSDV), bovine viral diarrhea virus (BVDV), bovine herpesvirus type 1 (BoHV-1), and bovine parainfluenza virus type 3 (BPIV-3), thereby providing a more comprehensive assessment of the broad-spectrum potential of BoIFN Acons. Additionally, the detailed molecular mechanisms of IFNAR binding and JAK-STAT signaling were not fully explored; further investigations into these pathways and the potential of BoIFN-Acons to modulate adaptive immune responses would be valuable. Despite these caveats, our study provides a safe, eco-friendly, orally deliverable interferon platform that offers a promising new strategy for controlling bovine viral diseases, with potential applicability to other major viral pathogens such as BVDV and FMDV.

## 5. Conclusions

In summary, this study systematically characterized the functional diversity of bovine interferon-alpha subtypes and identified BoIFN-A8 as the most potent natural variant. Through consensus sequence design, we further engineered a novel recombinant interferon, BoIFN-Acons, which exhibited significantly superior antiviral activity compared to all natural subtypes. By leveraging a food-grade *L. lactis* expression system, we developed a safe and orally deliverable platform that enables effective production and administration of BoIFN-Acons. Both in vitro and in vivo experiments demonstrated that this engineered strain potently suppresses viral replication, induces robust expression of interferon-stimulated genes, and confers significant prophylactic and therapeutic effects against viral infections in a mouse model. While these findings provide a promising proof-of-concept for an eco-friendly, orally deliverable antiviral strategy, we acknowledge that further validation in the target species (cattle) against clinically relevant viral pathogens is essential before clinical translation. Nevertheless, our work provides new insights into the subtype-specific functions of bovine IFN-α and establishes a foundation for the development of next-generation immunotherapeutics against viral diseases in livestock.

## Figures and Tables

**Table 1:** Overview of the experimental design.

Group	*n*	Pre-Challenge Treatment (Day −5 to −1)	Challenge (Day 0)	Post-Challenge Treatment (Day +1 to +5)
Blank control	6	PBS (100 μL)	PBS (100 μL)	PBS (100 μL)
VSV control	6	PBS (100 μL)	VSV (10^6^ TCID_50_ in 100 μL)	PBS (100 μL)
Recombinant prevention	6	pNZ-BoIFN-Acons/NZ3900 (1 × 10^9^ CFU in 100 μL)	VSV (10^6^ TCID_50_ in 100 μL)	PBS (100 μL)
Empty-vector prevention	6	pNZ8149/NZ3900 (1 × 10^9^ CFU in 100 μL)	VSV (10^6^ TCID_50_ in 100 μL)	PBS (100 μL)
Recombinant treatment	6	PBS (100 μL)	VSV (10^6^ TCID_50_ in 100 μL)	pNZ-BoIFN-Acons/NZ3900 (1 × 10^9^ CFU in 100 μL)
Empty-vector treatment	6	PBS (100 μL)	VSV (10^6^ TCID_50_ in 100 μL)	pNZ8149/NZ3900 (1 × 10^9^ CFU in 100 μL)

**Table 2:** The genome location of the BoIFN-α subtypes.

Subtype	Genomic Location	Length (bp)	Subtype	Genomic Location	Length (bp)
A1	22650137~22650706	570	A8	22952075~22952644	570
A2	22665782~22666378	597	A9	22998965~22999534	570
A3	22707500~22708069	570	A10	23029295~23029864	570
A4	22754645~22755214	570	A11	23054537~23055235	699
A5	22782695~22783264	570	A12	23073831~23074401	571
A6	22830867~22831436	570	A13	23125755~23126324	570
A7	22907850~22908419	570			

**Table 3:** Antiviral activity of BoIFN-A1/3/5/6/7/8/9/13/Acons.

Subtype	Antiviral Activity (10^2^ U/mg) (Mean + SD)
BoIFN-A1	2.23 + 0.75
BoIFN-A3	66.61 + 14.08
BoIFN-A5	45.79 + 14.56
BoIFN-A6	97.47 + 21.82
BoIFN-A7	8.79 + 1.53
BoIFN-A8	145.51 + 21.08
BoIFN-A9	1.88 + 0.58
BoIFN-A13	7.50 + 1.25
BoIFN-Acons	2357.17 + 944.24

## Data Availability

All the data that support the findings of this study are available from the corresponding author upon reasonable request.

## Supplementary Materials

The following supporting information can be downloaded at: https://www.mdpi.com/article/10.3390/ani16152327/s1, Figure S1: Nucleotide sequence alignment of various BoIFN-α subtypes and BoIFN-Acons; Table S1: Primer sequence; Table S2: Primer sequence.
