# Supplementary material for: A High-Potency Bovine Consensus Interferon Delivered by an Oral Lactococcus lactis System Exhibits Antiviral Activity Against VSV and BEV
Source: Animals (Basel). 2026 Jul 30;16(15):2327. doi: 10.3390/ani16152327 (PMC13463595; doi:10.3390/ani16152327)
Supplement: Supplementary file 1 [file animals-16-02327-s001.zip › animals-4378618-supplementary.pdf]

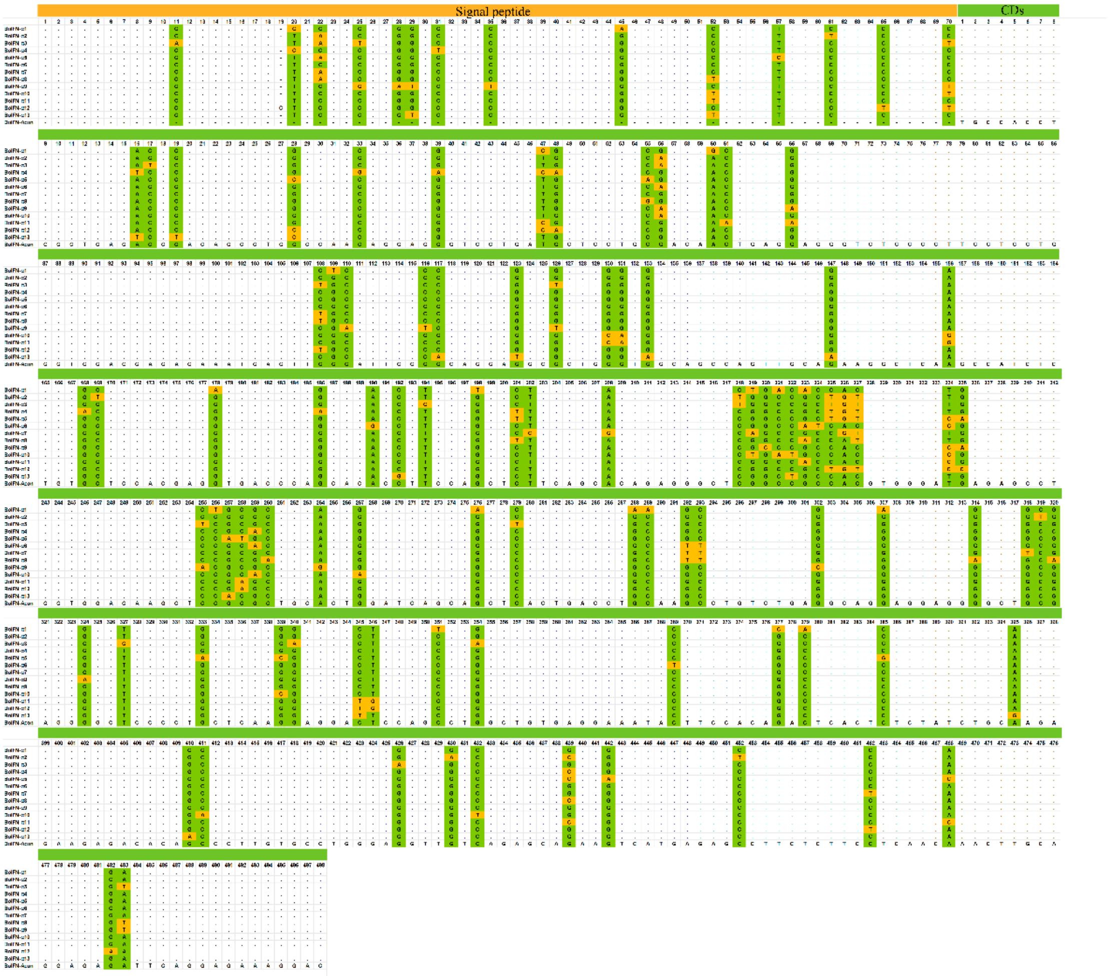

Figure S1. Nucleotide sequence alignment of various BoIFN-α subtypes and BoIFN-Acons. Conserved genes are denoted by black dots, while divergent genes are highlighted in yellow and green, respectively.

Table S1 Primer sequence

Primer Name	Sequence
1-F	TATGTTACATCAAAAGTCACTCTGAAAACTT
1-R	TCTGAAACCACTGTGTAAAGATTCACATATTATTA
3-F	CCATAGAGTCCATAAAAATATACCCAGTATACCTCT
3-R	TCAACTTTGGCATCTGTGAGGAACCAGTTCC
4-F	TAAGTAAATACCCTCCATCAAAAGTCAT
4-R	TCAGAAAATCCCCCCAAAATCCCCATTT
5-F	GAAGGACAACATGAAAAAAAAAAACAATG
5-R	AATTGAATCAATTTCACTGCATGATAACAGCT
6-F	GCAGAGCAATTAAGAAAAAAAT
6-R	TTGGCTTGGTGTTTCTGTTTT
7-F	GACTACTTCCATTCTCTATGTAT
7-R	CTTTATAAATAAGTAAATAAATAAATGTCC
8-F	GGTCAATAATGAAAACAACGTTTGCA
8-R	GAGAAAATCCCCAACACTTGTAATGTGGAAATCAC

9-F	ATGTACAGAGAAAAGATATCCATAGAGTACAT
9-R	TTAAATTAAGTTGCCATTCAGGATTGATGT
10-F	GATTCTCAACTCTACATTGAATACAAAAGTAATGT
10-R	TGTAACGACCAAGTGCCTATAGAGTCAT
13-F	TGGTCGGGGTAATAGGAAAAAAAATAT
13-R	AAATTCTTTAATCAAGTTGTGCATTCACCTCGG

Table S2 Primer sequence

Primer Name	Sequence
1-F	CGGGATCCTGCCACCTGCCTCACACC
1-R	CCCTCGAG**TCA**CTTGTCATCGTCGTCCTTGTAATCGTCCTTTCTCCTGAATCTCTCCT
3-F	CGGGATCCTGCCACCTGCCTCACATCC
3-R	CCCTCGAG**TCA**CTTGTCATCGTCGTCCTTGTAATCGTCCTTTCTCCTGAAACTCT
4-F	CGGGATCCTGCCACCTGCCTCACTCC
4-R	CCCTCGAG**TCA**CTTGTCATCGTCGTCCTTGTAATCGTCCTTTCTCCTGAATCTCTCCT
5-F	CGGGATCCTGCCACCTGCCTCACACC
5-R	CCCTCGAG**TCA**CTTGTCATCGTCGTCCTTGTAATCGTCCTTTCTCCTGAATCTCTCCT
6-F	CGGGATCCTGCCACCTGCCTCACACC
6-R	CCCTCGAG**TCA**CTTGTCATCGTCGTCCTTGTAATCGTCCTTTCTCCTGAATCTCTCCT
7-F	CGGGATCCTGCCACCTGCCTCACACC
7-R	CCCTCGAG**TCA**CTTGTCATCGTCGTCCTTGTAATCGTCCTTTCTCCTGAATCTCTCCT
8-F	CGGGATCCTGCCACCTGCCTCACACC
8-R	CCCTCGAG**TCA**CTTGTCATCGTCGTCCTTGTAATCGTCCTTTCTCCTGAAACTCT
9-F	CGGGATCCTGCCACCTGCCTCACACC
9-R	CCCTCGAG**TCA**CTTGTCATCGTCGTCCTTGTAATCGTCCTTTCTCCTGAAACTCT
10-F	CGGGATCCTGCCACCTGCCTCACACC
10-R	CCCTCGAG**TCA**CTTGTCATCGTCGTCCTTGTAATCGTCCTTTCTCCTGAATCTCTCCT
13-F	CGGGATCCTGCCACCTGCCTCACTCCT
13-R	CCCTCGAG**TCA**CTTGTCATCGTCGTCCTTGTAATCGTCCTTTCTCCTGAATCTCTCCT

Note: The underlined portion indicates the restriction site, and the bold portion indicates the terminator.
